# Quantification of gray mold infection in lettuce using a bispectral imaging system under laboratory conditions

**DOI:** 10.1002/pld3.317

**Published:** 2021-03-24

**Authors:** Clifton G. Scarboro, Stephanie M. Ruzsa, Colleen J. Doherty, Michael W. Kudenov

**Affiliations:** ^1^ Department of Electrical and Computer Engineering Optical Sensing Laboratory North Carolina State University Raleigh NC USA; ^2^ Department of Molecular and Structural Biochemistry North Carolina State University Raleigh NC USA

**Keywords:** disease detection, gray mold, hyperspectral imaging, multispectral imaging, plant pathology, reflectance indexing

## Abstract

Gray mold disease caused by the fungus *Botrytis cinerea* damages many crop hosts worldwide and is responsible for heavy economic losses. Early diagnosis and detection of the disease would allow for more effective crop management practices to prevent outbreaks in field or greenhouse settings. Furthermore, having a simple, non‐invasive way to quantify the extent of gray mold disease is important for plant pathologists interested in measuring infection rates. In this paper, we design and build a bispectral imaging system for discriminating between leaf regions infected with gray mold and those that remain unharmed on a lettuce (*Lactuca* spp.) host. First, we describe a method to select two optimal (high contrast) spectral bands from continuous hyperspectral imagery (450–800 nm). We then explain the process of building a system based on these two spectral bands, located at 540 and 670 nm. The resultant system uses two cameras, with a narrow band‐pass spectral filter mounted on each, to measure the bispectral reflectance of a lettuce leaf. The two resulting images are combined using a normalized difference calculation that produces a single image with high contrast between the leaves’ infected and healthy regions. A classifier was then created based on the thresholding of single pixel values. We demonstrate that this simple classification produces a true‐positive rate of 95.25% with a false‐positive rate of 9.316% in laboratory conditions.

## 1 INTRODUCTION

**FIGURE 1:**
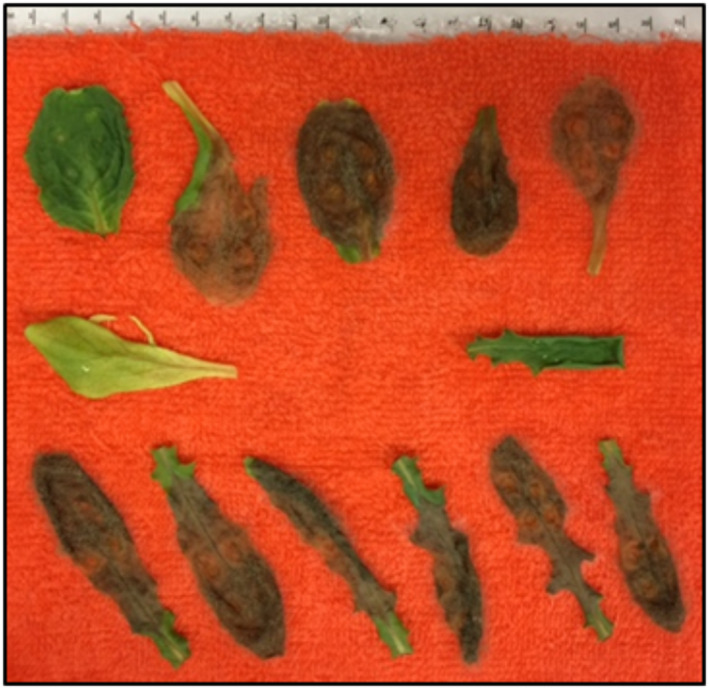
Salinas (top row) and US96 (bottom row) lettuce leaves 7 days after inoculation with *Botrytis cinerea* in a controlled temperature chamber. The middle row of leaves contains uninoculated control Salinas (left) and US96 (right) leaves

*Botrytis cinerea* is an airborne plant pathogen that causes losses by means of gray mold disease in over 200 different species of crops worldwide, with the most damage being seen in dicotyledonous hosts (Williamson et al., 2007). Typical symptoms of the disease observed in plant leaves and fruits include soft rots followed by the appearance of gray masses of conidia (Williamson et al., 2007). A representative color image of various Salinas and US96 lettuce (*Lactuca sativa*) leaves with severely advanced infection is shown in Figure 1.

Gray mold is also a major threat to greenhouse‐grown plants, where control of the disease is usually challenging and expensive (Dik & Elad, 1999). Careful application of fungicides would allow for effective management of gray mold when growing plants. Using fewer well‐timed sprays of a fungicide is more efficient than more frequent applications through harvest due to the possibility of promoting resistant populations of *B. cinerea* (Wedge et al., 2007). An imaging system that can highlight areas of gray mold infection in plant leaves with accuracy and consistency, ideally before symptoms are visible, would allow for quick and easy assessment of the disease on a large number of plants at once. In this way, growers can make informed decisions on spraying fungicide. In addition, as demonstrated here, such an imaging system provides a useful tool for plant pathologists studying the spread of gray mold. The ability to consistently and quantitatively detect the first signs of *B. cinerea* and to monitor disease progression in a leaf can provide detailed trait information to researchers comparing treatment methods or investigating genotype‐driven differences in susceptibility. Several studies have addressed gray mold diagnosis and detection in plants. Traditionally, fungal disease‐causing pathogens have been detected by visual examination of symptoms on the plant (Ray et al., 2017). This involves interpreting disease symptoms such as blight, rotting, spots, and wilting using available guidelines and standards for assessment (Ray et al., 2017). Some drawbacks to this method are that its effectiveness is subject to an examiner's judgment and therefore produces variable results. Scoring by eye is also time‐consuming and can be difficult to scale for more high‐throughput analysis or high‐resolution temporal analysis throughout the infection progression. Molecular quantification based on quantitative polymerase chain reaction or counting fungal conidiospores offers increased throughput (Weßling & Panstruga, 2012). However, these methods are destructive and therefore, the same individual plant cannot be monitored across time. Additional detection methods include plating and culturing plant pathogens for identification using microscopy techniques (Narayanasamy & Narayanasamy, 2011), but these approaches are challenging to scale for high‐throughput, high‐resolution temporal analysis. Less time‐consuming methods for detecting the extent of infection in a plant population involve spectroscopic and imaging techniques. Fungal diseases can alter leaf morphology, and therefore their light reflectance, which can be distinguished using hyperspectral or multispectral imaging (Fahrentrapp et al., 2019). Fahrentrapp et al. (2019) demonstrated that measuring the reflectance of a leaf at narrow‐band near‐infrared (NIR) wavelengths was sufficient to distinguish between a leaf's healthy and infected areas using a linear regression model. An issue with the model used is that it requires taking into account the relative position between the leaf and detector, as well as the lighting conditions. Wu et al. (2008) used a hyperspectral visible near‐infrared (VNIR) spectroradiometer to measure reflectance intensities of healthy and *B. cinerea*‐inoculated eggplant leaves. By performing a principal component analysis on the collected data and training a back‐propagation neural network on this data, the authors were able to predict infection with 85% accuracy before infection symptoms were visible. The results relied upon collecting data in a controlled environment where ambient lighting was limited. However, while such VNIR approaches may offer improved early detection, they are more expensive and complex to deploy in automated imaging applications. Spectral angle mapping (SAM), where reference spectra are compared to measured spectra by determining their angular differences when treated as n‐dimensional vectors, has also been used with decent success to quantify disease spread in plants. Mahlein et al. (2012) analyzed *Cercospora* leaf spot, powdery mildew, and leaf rust on sugar beets using SAM which achieved a classification accuracy of 89.01%–98.90% depending on the severity of the disease. Two‐band detection methods have also been tested. Mo et al. (2015) found optimal wavebands at 552 and 701 nm in a two‐band ratio for detecting discoloration in Salinas lettuce leaves with over 99% accuracy; however, its application towards *Botrytis* detection was not studied.

In this paper, we demonstrate a process for selecting optimal spectral bands using hyperspectral imaging. These bands are then used in a bispectral imaging system to acquire a normalized difference index. We evaluate this index's performance in detecting *B. cinerea* infection on *L. sativa* leaves to monitor disease progression across two diverse genotypes. In section 2, we outline the theory needed to assess the hyperspectral data as well as our experimental setup. Section 3 describes the results of our hyperspectral data as well as the bispectral imaging system across time lapse data. Section 4 provides a discussion of the results of the bispectral imaging system.

## 2 MATERIALS AND METHODS

### 2.1 Spectral band optimization

Hyperspectral and multispectral imaging devices have been widely used for measuring irradiance spectra of a scene with varying spectral resolution, range, and acquisition methods. They measure light collected from a scene as a function of two spatial dimensions and one spectral dimension. The main distinction between a hyperspectral and multispectral system is the number of spectral bands measured by the device. There are a variety of methods for acquiring this three‐dimensional data that are mostly described by the way they discriminate the spatial and spectral dimensions (Sellar & Boreman, 2005). Examples of spatial data acquisition methods include whiskbroom (point‐scanning) and pushbroom (line‐scanning) systems, as well as “snapshot” two‐dimensional imagers (Sellar & Boreman, 2005). Some ways these devices acquire spectral data include spectral filtering, dispersive elements like prisms or diffraction gratings, and interferometric Fourier transform methods (Sellar & Boreman, 2005).

To discover the optimal spectral bands, we use a prism‐based pushbroom scanning hyperspectral imaging camera that has been calibrated per Ref. Kudenov et al. (2017) to provide comprehensive lettuce irradiance spectra. Relative reflectance was calculated to quantify both healthy and infected lettuce leaf tissues, such that

(2.1) $$\rho \left(x_{n},y_{m},\lambda_{k}\right)=I_{\mathrm{sample}}\left(x_{n},y_{m},\lambda_{k}\right)/I_{\mathrm{tile}}\left(y_{m},\lambda_{k}\right),$$

where *I_sample_* is the intensity measured from the lettuce, *I_tile_* is the downwelling intensity measured from a white (99% reflectivity) spectralon tile (Labsphere, SRT‐99‐050) illuminated by the source. *x_n_* and *y_m_* are the *x*‐ and *y*‐coordinates corresponding to the discrete integer spatial position *n* and *m*, respectively, and *λ_k_* is the *k*th wavelength element in the data cube. For visualization and labeling purposes, two‐dimensional (2D) panchromatic imagery was generated by integrating our three‐dimensional (3D) data cube along the spectral axis for each image slice such that

(2.2) $$I_{P}\left(x_{n},y_{m}\right)=\sum_{k=0}^{K}\rho \left(x_{n},y_{m},\lambda_{k}\right).$$

After the reflectivity has been calculated, we leveraged graphical reflectance indexing to provide a simple analysis of the scene's spectral measurements. By taking radiance measurements at specific spectral components, correlations between features of the scene can be made. A common example of this is the normalized difference vegetation index (NDVI) which takes the difference of reflectance of two spectral bands in the near‐infrared (NIR) and red regions and divides by their sum (Carlson & Ripley, 1997). Generally, the normalized difference reflectance index, *ν*, from such two‐band calculations for arbitrary wavelengths, *λ_1_* and *λ_2_*, is described by

(2.3) $$\nu \left(\lambda_{1},\lambda_{2}\right)=\frac{I\left(\lambda_{1}\right)‐I\left(\lambda_{2}\right)}{I\left(\lambda_{1}\right)+I\left(\lambda_{2}\right)},$$

where the intensity measurements *I* are implicitly dependent upon *x_n_* and *y_m_* for clarity. This procedure reduces the effect of lighting and atmospheric conditions on measurements to enable more accurate comparison (Rouse, 1974). Also, image data from two spectral bands can be condensed into one single image that is produced from the contributions of both bands.

**FIGURE 2:**
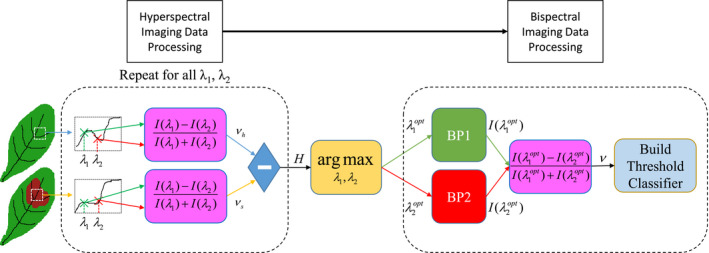
Overall work pipeline. Hyperspectral data are collected for healthy and symptomatic lettuce leaves, and the normalized difference reflectance indices corresponding to each type, *ν_h_* and *ν_s_*, are calculated for each spectral band combination, *λ*_1_ and *λ*_2_, using Equation (2.3). Additionally, the absolute difference is taken between the indices for each type to produce the heat map, *H*, from Equation (2.4). Following Equation (2.5), the maximum difference in the heat map is used to select the spectral bands, *λ*_1_ *^opt^* and *λ*_2_ *^opt^*, that are utilized in the bispectral imaging system in the band‐pass filters, BP1 and BP2. Each of the band‐pass images, *I*, are used to calculate the normalized difference reflectance index, *ν*, using Equation (2.3) and further statistical analysis is performed based on this resulting image

The goal for a system implementing reflectance indices is to select two spectral bands where the contrast is optimized between two characteristics of interest, *a* and *b* (*e.g*., symptomatic versus healthy areas of lettuce leaves). Using data from the hyperspectral reflectance plots, a heat map, which is defined by

(2.4) $$H\left(\lambda_{1},\lambda_{2}\right)=\left|\nu_{a}\left(\lambda_{1},\lambda_{2}\right)‐\nu_{b}\left(\lambda_{1},\lambda_{2}\right)\right|,$$

was obtained by sweeping through each combination of two spectral bands, *λ_1_* and *λ_2_*, as inputs to Equation (2.3) to calculate the contrasts, *ν_a_* and *ν_b_*, associated with the characteristics, *a* and *b*. By locating the maximum in *H* as defined by

(2.5) $$\lambda_{1}^{\mathrm{opt}},\lambda_{2}^{\mathrm{opt}}=\underset{\lambda_{1},\lambda_{2}}{\arg\max}\left(H\right),$$

the optimal spectral bands, *λ*_1_ *^opt^* and *λ*_2_ *^opt^*_,_ can be determined for use in a bispectral camera system. The overall procedure for determining *λ*_1_ *^opt^* and *λ*_2_ *^opt^* for use in the bispectral camera system with the purpose of classifying healthy and symptomatic lettuce leaf areas is depicted in a workflow diagram in Figure 2.

### 2.2 Lettuce and *B. cinerea* Growth and Inoculation

*Lactuca sativa* cv. “Black Seeded Simpson” (Black Seeded Simpson), *Lactuca sativa* cv. “Salinas” (Salinas), and *Lactuca serriola* acc. US96UC23 (US96) seeds were imbibed in sterile deionized water, protected from light. After 72 hr, seeds were plated on half‐strength MS (Murashige & Skoog, PhytoTechnology Labs) salts, 0.4% plant tissue culture agar (PhytoTechnology Labs), pH 5.7. At seven days, seedlings were transplanted, at one seedling per pot, to four‐inch plastic pots filled with Redi‐Earth Seed Mix (Wyatt Quarles), moistened with tap water. Growing plants were watered from below as needed with tap water. Both plates and pots were kept in growth chambers under 12‐hr light, 12‐hr dark cycles, at 22°C days and 19°C nights.

*Botrytis cinerea*, provided by Inga Meadows (NCSU Entomology & Plant Pathology Extension Associate, Mountain Research station AtW 1, Waynesville, NC), was maintained on Potato Dextrose Agar (PDA) plates (PhytoTechnology Labs) in 12‐hr light, 12‐hr dark cycles at a constant 23°C. To produce spores for inoculation, *Botrytis* was grown on either PDA or V8 plates (20% V8 juice, 0.2% calcium carbonate, 1.5% bacteriological agar [Fisher Scientific]). Spore suspensions were made from vigorously growing (9‐ to 21‐day old) plates by washing and resuspending the spores with sterile inoculating loops in 10–15 ml sterile water. Suspensions were filtered through four layers of sterile cheesecloth and stirred gently for 15–20 min, with 0.1% Tween 20 (Sigma). Spores were counted in a Brandt, Blaubrand, Neubauer improved, Brightline Hemacytometer (Sigma‐Aldrich) on a Zeiss Axioplan microscope and diluted with sterile Potato Dextrose Broth (PDB, PhytoTechnology Labs) to 1 × 10^−5^ spores/ml.

Lettuce leaves were detached from 16‐ to 21‐day post‐transplanted lettuce plants. Leaves were chosen from newer growth (5–7 days after emergence), for the similarity of size and growth stage. Detached leaves were placed and kept in a plastic, sealable bag until ready for use, but not more than 30 min. Initially, paper towels were laid out in 254‐mm Square BioAssay Dishes (Corning) and moistened with sterile deionized water. For better imaging contrast in the dual‐band imaging system, red‐hued terry cloth (Room Essentials: Dreamy Tangerine, Target) was subsequently used. Lettuce leaves were laid out on the moistened material and inoculated with the diluted spore suspensions using a pipette at 5 µl per inoculation. Each leaf was inoculated four times, twice on either side of the midvein. Control leaves were mock‐inoculated with sterile PDB. Plates were returned to the chamber under lettuce growth conditions as above. For time lapse imaging, where the plants were not returned to the chamber, the detached lettuce leaves were placed in a 100‐mm square Petri dish on moistened terry cloth, inoculated with *Botrytis*, and kept at room temperature (~23°C) in constant light.

The Black Seeded Simpson variety was used with the hyperspectral imaging system to identify the spectral bands of interest. Salinas and US96 genotypes were tested using the optimized bispectral imaging system to determine the ability to monitor the affected areas in both genotypes.

### 2.3 Experimental setup and data processing

**FIGURE 3:**
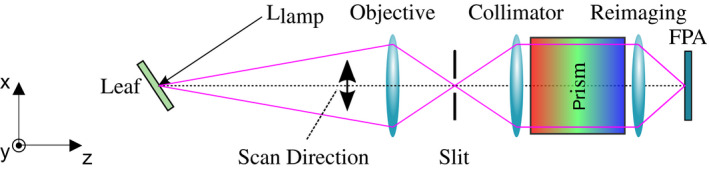
Hyperspectral pushbroom camera for preliminary characterization of lettuce infected with *Botrytis cinerea*

To calculate *v* over many combinations of spectra, a pushbroom hyperspectral imaging camera was used to collect a three‐dimensional spatial and spectral data cube over a visible to near‐infrared spectral range with wavelength resolution spanning 0.5–2.4 nm for 500–800 nm light. Lettuce leaves of the variety Black Seeded Simpson were inoculated with *B. cinerea*, which spread throughout the leaves over 3–5 days. When the disease symptoms were significant (about 50% symptom coverage of the leaf area) on most inoculated lettuce leaves, the tray was imaged by the pushbroom camera, and a tungsten light source was used to illuminate the sample leaves. The pushbroom camera and tungsten light source setup are presented in Figure 3. A tungsten halogen lamp was configured to illuminate the target at a normal angle of incidence from the leaf's surface normal. Our pushbroom spectral camera was positioned 40 cm away, yielding a spatial resolution of approximately 6.5 mm. Light entered the objective lens, slit, and collimator where it was dispersed using a prism. Dispersed light was then imaged onto a focal plane array (FPA, Allied Visions Manta G‐033B) using a reimaging lens and raw data were calibrated in accordance with Ref. Kudenov et al. (2017). The FPA measures an image with a spatial dimension along the slit and a spectral dimension caused by the dispersive prism. To acquire the other spatial dimension required for a two‐dimensional image, the entire imaging system is mounted on a rotating platform that moves while the camera acquires 1,200 successive frames that form the three‐dimensional data cube, *I_sample_*. This process takes 2 min to scan over a 120° field of view.

After acquiring the hyperspectral data cubes, two‐dimensional images of the lettuce were obtained using Equation (2.2). Infected and control (healthy) tissues, from inoculated and control leaves, respectively, were manually labeled by defining regions of interest (ROIs) of 10 x 10 pixels that were selected by comparing to RGB images scored by trained individuals. The boundaries of the affected regions were avoided. Each ROI was then spatially averaged to create normalized reference spectra. These reference spectra are then used to produce a heat map by sweeping through all spectral bands in calculating Equation (2.4). We find the optimal spectral bands for our dual‐camera bispectral system are the two bands that produce the highest contrast in this heat map. Spectral band‐pass filters centered at these wavelengths can then be acquired.

**FIGURE 4:**
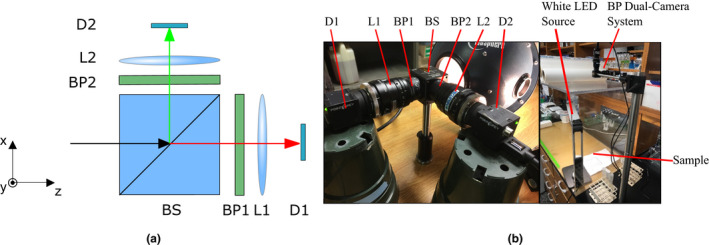
Band‐pass filtered dual‐camera system (a) schematic and (b) labeled photographs

A schematic of the two‐band imaging system is depicted in Figure 4a. Light from the sample first enters a beam splitter (BS), which directs light from a scene to each camera. Transmitted or reflected light from the BS then enters the spectral band‐pass filters, BP1 or BP2, respectively, before forming an image by L1 or L2 onto the FPAs, D1 or D2, respectively. The FPAs consisted of two BlackFly 1.3 megapixel, 30 frames per second USB3 monochrome cameras (FLIR Systems, BFLY‐U3‐13S2M‐CS). Both cameras were configured to take images of the scene simultaneously. A photograph of the completed experimental setup is depicted in Figure 4b. The bispectral camera system was used to obtain contrast images of two distinct species—*Lactuca sativa* cv. Salinas and *L. serriola* acc. US96UC23 (US96), parents of a recombinant inbred mapping population (Truco et al., 2013). Since this system was used to take lettuce measurements over several days, a 7.6W white LED desk lamp (Prism TL‐4300) was used that does not radiate as much heat as the tungsten lamp. This helps mitigate environmental stress on the lettuce leaves. In addition, experiments were performed inside of a clear plexiglass container to prevent the spread of *B. cinerea* to the surrounding environment. Since the imaging system was located outside of the plexiglass barrier, glare off its surface was reduced by placing the LED off‐axis in an illumination geometry similar to darkfield microscopy (Villiger et al., 2010).

In post‐processing, an affine image registration algorithm (Bergen et al., 1992) aligns the two images, taking into account translational and rotational differences. In addition, both of these images are normalized to flat field images of the white spectralon tile taken prior to measurements with the same camera settings using Equation (2.1). This corrects for non‐uniform spectral radiance from the LED and differences in shutter time and aperture size in the cameras. Finally, the calculation from Equation (2.3) is applied using the spatially registered images as input arguments. To enable rapid segmentation of the individual leaf's boundaries while maintaining moisture, they were placed on a red terrycloth background. This provided a high positive value of *v* that could be easily distinguished from the diseased or healthy tissues’ lower values. The background pixel values are assigned to global minimum values during post‐processing so that the predominantly white leaves in the grayscale contrast images stand out better against a black background. By thresholding pixel values, very high values are assigned as belonging to the background.

Additional processing was implemented to measure the percent area of the lettuce leaves that were covered by the gray mold disease. Within each leaf, the diseased and healthy areas were segmented by further thresholding the pixel intensities. To determine a reasonable threshold to best discriminate between healthy and symptomatic lettuce areas, approximately 400 pixels belonging to each disease classification were labeled by visual inspection in a set of contrast images to compare pixel values. Using the symptomatic and healthy pixel means (*μ_s_* and *μ_h_*) and standard deviations (*σ_s_* and *σ_h_*), a decision threshold, *η*, was defined by estimating a probability density function (PDF) for each type and finding *η* such that

(2.6) p(η|H)=p(η|S),

where ***H*** is the event that a pixel belongs to a healthy lettuce leaf, and ***S*** is the event that a pixel belongs to a symptomatic leaf.

## 3 RESULTS

### 3.1 Hyperspectral reflectivity characterization

**FIGURE 5:**
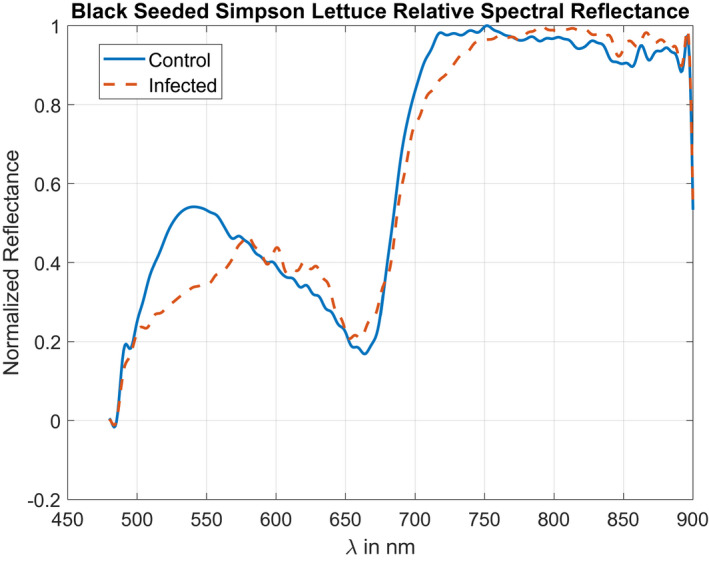
Normalized spectral response measured for symptomatic and control lettuce leaves of the Black Seeded Simpson variety. Based on these plots, the spectral band‐pass filters were chosen with center pass‐bands at 540 and 670 nm

The normalized spectral reflectivity plots for the Black Seeded Simpson lettuce variety are depicted in Figure 5 for healthy and diseased conditions. Generally, the normalized reflectivity is the same except around 540 and 720 nm. Our reflectance measurements presented in Figure 5 are supported by reflectivity measurements taken by Simko et al. (2015) for lettuce tissue in fresh and decayed conditions.

**FIGURE 6:**
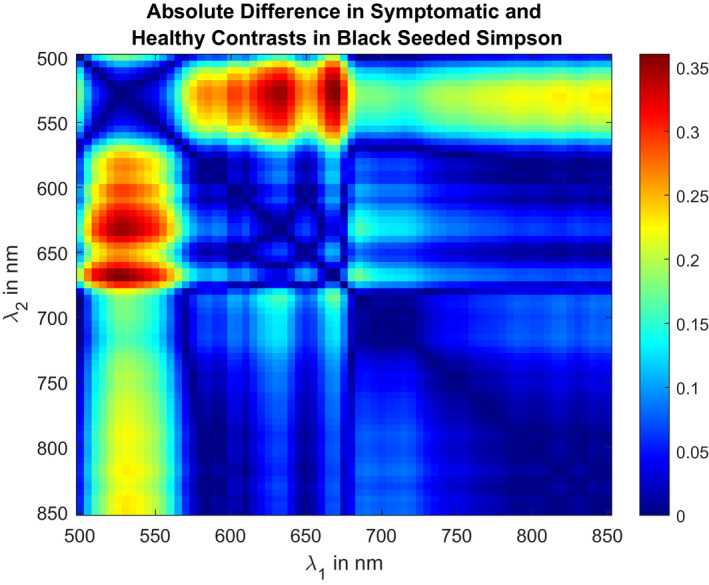
Heat map of the absolute difference in contrasts of two spectral bands measured on healthy and symptomatic lettuce areas for the Black Seeded Simpson type

These reference spectra were further used to produce the heat map in Figure 6. It should be noted that the heat map is symmetric about the diagonal due to the same spectral bands used in the calculation from Equation (2.4). From these results, the highest contrast is located at *λ*_1_ = 670 nm and *λ*_2_ = 530 nm. Using this information, we selected two spectral band‐pass filters for use in a dual‐camera bispectral system. The filters selected had center pass‐bands at 540 nm and 670 nm and a 10 nm full width at half maximum spectral bandwidth. Note that one filter (540 nm) deviates slightly from our calculated maximum due to off‐the‐shelf filter availability at the time of purchase; however, this is only expected to reduce *v* by 2.8%.

### 3.2 Bispectral imaging system performance

**FIGURE 7:**
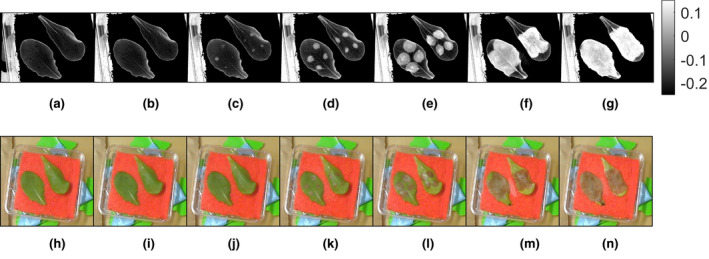
Contrast images (v) and color reference images taken over 6 days at roughly the same time each day of Salinas (left in images) and US96 (right in images) lettuce varieties inoculated with *Botrytis cinerea*. Each contrast image corresponds to the number of days after inoculation: (a) Day 0, (b) Day 1, (c) Day 2, (d) Day 3, (e) Day 4, (f) Day 5, and (g) Day 6. Each color reference image also corresponds to a day after inoculation: (h) Day 0, (i) Day 1, (j), Day 2, (k) Day 3, (l) Day 4, (m) Day 5, and (n) Day 6

To examine the ability to identify *Botrytis cinerea*‐affected regions in diverse lettuce backgrounds, we applied this approach to *L. sativa* cv. Salinas and *L. serriola* acc. US96UC23, the parents of a recombinant inbred population (Truco et al., 2013). *L serriola* is likely the wild progenitor to L. sativa and the two genotypes have distinct pigmentation differences (Lindqvist, 1960). Figure 7 shows the contrast images of a US96 and Salinas leaf over 7 days alongside a reference color image taken using a color USB camera. The healthy lettuce areas have lower values of *v* when compared to the damaged areas. This is expected from our hyperspectral results since healthy tissue yields a lower reflectance at 670 nm compared to 540 nm (producing a negative value using Equation (2.3)). In contrast, infected tissue has a more similar reflectivity at both wavelengths (producing a positive value closer to 0). In both genotypes, the wavelengths consistently distinguished between healthy and affected tissues.

**FIGURE 8:**
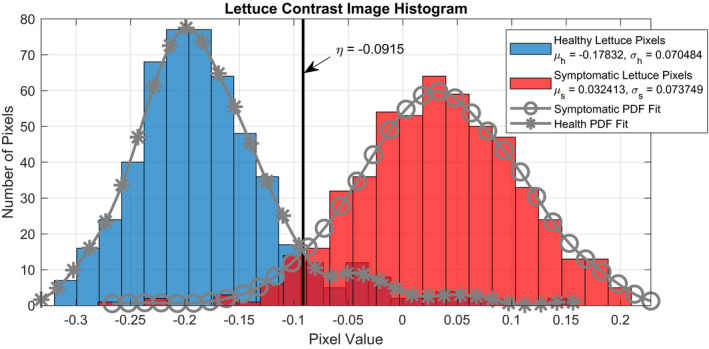
Histogram of pixel values corresponding to healthy and symptomatic lettuce areas compiled for both US96 and Salinas varieties. A threshold for discriminating between the two classifications was determined using these distributions

**FIGURE 9:**
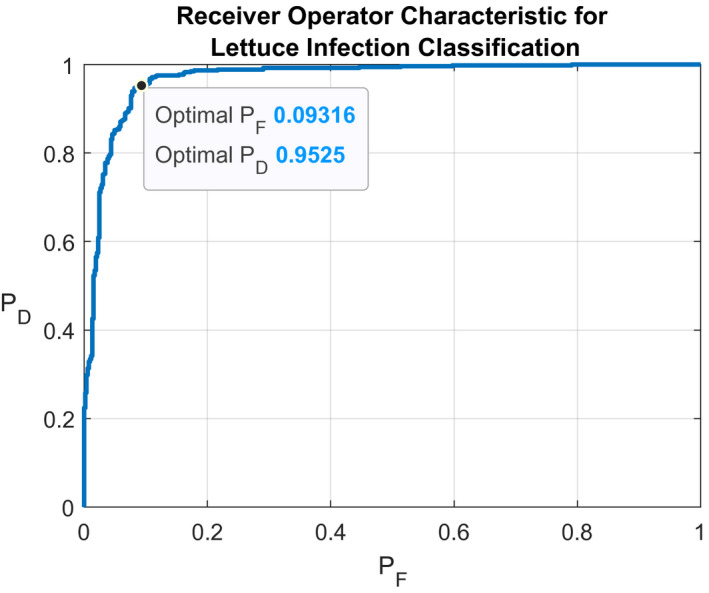
Receiver Operating Characteristic (ROC) for classifier based on thresholding band‐pass contrast images of infected lettuce leaves. P_F_ is the false‐positive disease detection probability, while P_D_ is the true‐positive disease detection probability. The area under the curve is 0.9698

Histograms of *v* for both the healthy and symptomatic pixel classes are presented in Figure 8. Based on the fitted PDFs, a contrast threshold of *η* = −0.0915 was found to be sufficient for discriminating healthy and symptomatic tissues. In Figure 9, a receiver operating characteristic (ROC) curve was created based on the aforementioned PDFs. From this curve, it can be seen that for a probability of false‐positive classification, *P_F_*, of symptomatic tissue equal to 0.09316, the true‐positive probability, *P_D_*, is 0.9525, which yields a classification accuracy of 0.92967.

**FIGURE 10:**
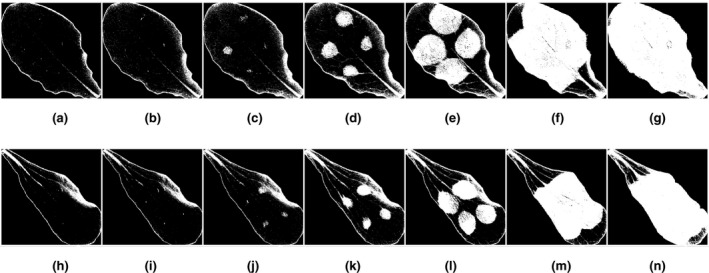
Binary classification of time lapse images shown in Figure 7. Using the determined threshold, *η* = −0.0915, Salinas and US96 leaves are presented where white areas indicate disease spread detected while black areas indicate no symptoms detected. The classification decision was made by comparing the contrast images from the band‐pass dual‐camera system to *η*. Images are sequenced by days after *B. cinerea* inoculation for Salinas leaves on (a) Day 0, (b) Day 1, (c) Day 2, (d) Day 3, (e) Day 4, (f) Day 5, and (g) Day 6, and for US96 leaves on (h) Day 0, (i) Day 1, (j) Day 2, (k) Day 3, (l) Day 4, (m) Day 5, and (n) Day 6

A binary classification image that uses this decision threshold is depicted in Figure 10. Some false‐positive areas in the binary imagery are identified as belonging to a diseased lettuce region when they are not. These areas occur around the leaves’ edges where the red background was classified as part of the leaf due to shadowing effects and was not removed from the image, and along the leaf veins, which can tend to have more yellow coloration than the leaf blades. To account for this when calculating the severity of disease symptoms on a particular leaf, an offset subtraction described by

(3.1) $$N_{net,s}\left(t\right)=N_{raw,s}\left(t\right)‐N_{raw,s}\left(t_{0}\right),$$

where *N_raw,s_*(*t*) is the number of pixels classified as symptomatic at time, *t*, and *t_0_* is the time of the first binary image immediately after inoculation. *N_net,s_*(*t*) is the net number of symptomatic pixels in the binary image after *t* hours. On Day 2 of the experiment, the first signs of infection are observed and classified.

**FIGURE 11:**
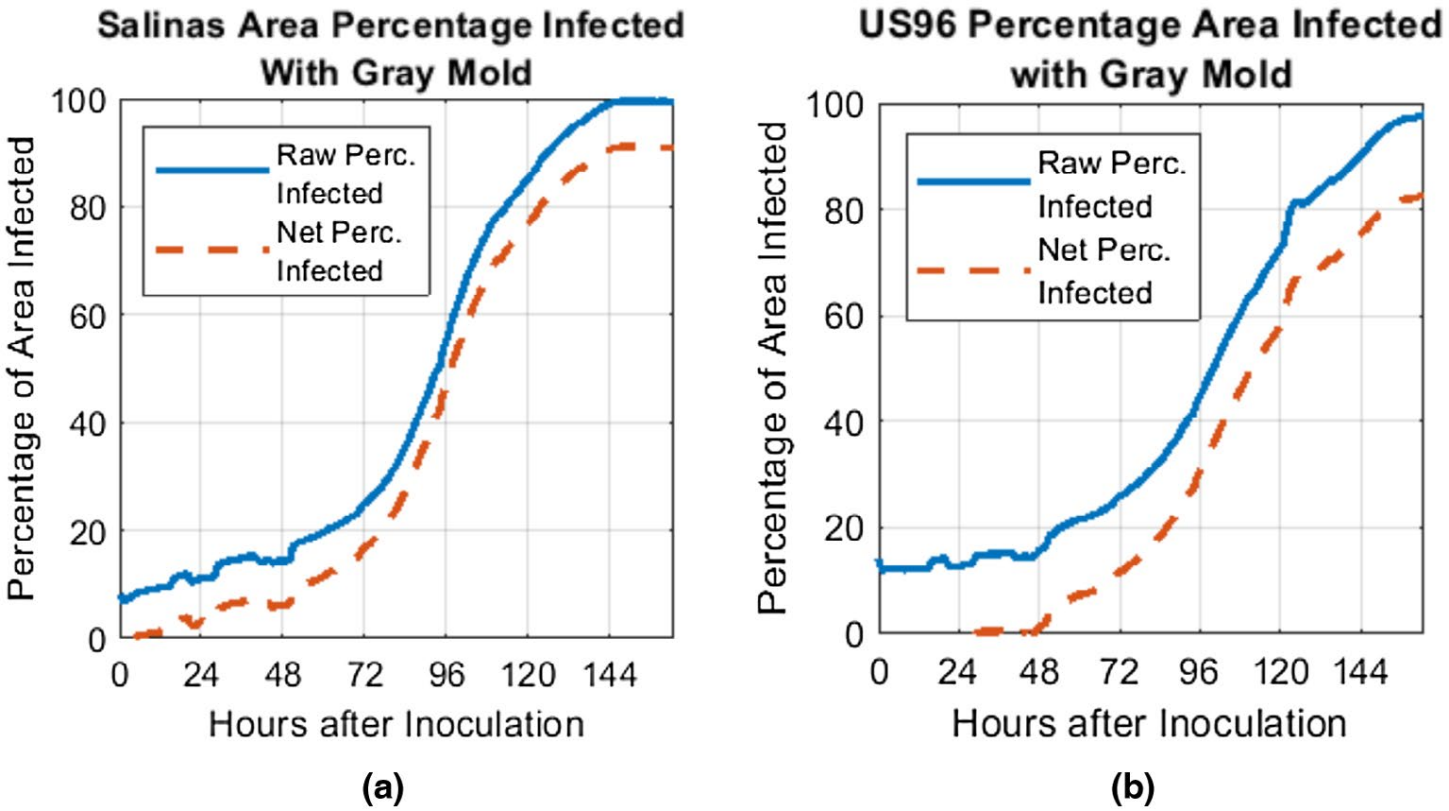
Plot of percentage leaf area infected with gray mold for (a) Salinas and (b) US96 types. These quantities were calculated by taking a ratio of the number of disease‐classified pixels to the entire leaf area based on the binary images in Figure 10

The diseased leaf area ratio to the total leaf area was calculated and presented as a percentage in Figure 11a,b for Salinas and US96, respectively. Both the diseased area percentage with and without offset subtraction in Equation (3.1) are provided in the plots. This provides a data metric for studying the infection's spread and leaf damage over time. According to these plots, the symptoms spread at what appears to be an exponential rate until the leaf is close to fully damaged. At this point, the rate of disease spread starts to decrease, consistent with a population growth curve approaching the carrying capacity.

## 4 DISCUSSION

Here we present a methodology for building a bispectral imaging system to locate gray mold infection in plant leaves. A pushbroom hyperspectral imaging system was leveraged to select two narrow spectral band‐pass filters to use in the dual‐camera system. Post‐processing of the collected data provides a basis for visually identifying infection areas and quantifying the spread over time. The normalized difference between the two band‐pass images produced a significant contrast between leaf areas with gray mold disease and unaffected areas with a simple computation. A threshold was used to programmatically discriminate between infected and healthy leaf areas with reasonable accuracy. The threshold we used in this paper was optimal in the sense that pixel values below the threshold have a higher likelihood of being symptomatic while above‐threshold pixel values are more likely to be healthy areas. This threshold could be altered to achieve different goals, such as an underestimation of the infection site size but with fewer false‐positive detections. While the results here are demonstrated only in indoor plants, this approach was developed to be implemented even in direct sunlight. A white reference tile can normalize the effects of different illumination sources like the Sun on the band‐pass images. However, care should be taken to ensure specular reflections between the light source and imaging system are minimized because excess surface reflections can obscure the volumetric reflections and lead to inaccurate calculations of *v*.

The underlying variation across different genotypes presents a challenge when using spectroscopic approaches to identify quantitative traits in a mapping or association population. Here, by combining the information from two wavelengths, 670 and 540 nm, we have developed a method that is robust to the pigment differences in different colorings of two species of lettuce, L. sativa “Salinas,” (Salinas) and L. serriola US96UC23 (US96). While hyperspectral imaging may provide better segmentation of the gray mold infection sites (Mahlein et al., 2012; Mo et al., 2015), our system provides reasonable segmentation with reduced data by only measuring two spectral bands as opposed to upwards of 25 spectral bands. We have achieved a classification accuracy comparable to other multispectral systems used for fungal disease detection (Fahrentrapp et al., 2019; Simko et al., 2015; Wu et al., 2008). This makes our system ideal for high‐throughput infection quantification for many different lettuce genotypes.

## AUTHORS’ CONTRIBUTIONS

M.W.K. and C.J.D. conceived the original research experiment. C.G.S. wrote the original manuscript with edits and revisions provided by M.W.K., C.J.D., S.M.R, and C.G.S. S.M.R. and C.J.D. grew the lettuce plants, generated the *B. cinerea* suspension, and established and implemented the procedure for inoculating the lettuce leaves with the suspension for the experiments. C.G.S. performed the calibration for, and collected and processed data from the hyperspectral pushbroom camera with guidance from M.W.K. C.G.S. constructed the dual‐camera band‐pass system and processed and analyzed the data acquired from the system with guidance from M.W.K.

## References

[pld3317-bib-0001] Bergen, J. R. , Anandan, P. , Hanna, K. J. , & Hingorani, R. (1992). Hierarchical model‐based motion estimation. In G. Sandini (Ed.), Computer Vision — ECCV’92, Lecture Notes in Computer Science (pp. 237–252). Springer.

[pld3317-bib-0002] Carlson, T. N. , & Ripley, D. A. (1997). On the relation between NDVI, fractional vegetation cover, and leaf area index. Remote Sensing of Environment, 62, 241–252.

[pld3317-bib-0003] Dik, A. J. , & Elad, Y. (1999). Comparison of antagonists of botrytis cinerea in greenhouse‐grown cucumber and tomato under different climatic conditions. European Journal of Plant Pathology, 105, 123–137.

[pld3317-bib-0004] Fahrentrapp, J. , Ria, F. , Geilhausen, M. , & Panassiti, B. (2019). Detection of gray mold leaf infections prior to visual symptom appearance using a five‐band multispectral sensor. Frontiers in Plant Science, 10, 628. 10.3389/fpls.2019.00628 31156683 PMC6529515

[pld3317-bib-0005] Kudenov, M. W. , Lowenstern, M. E. , Craven, J. M. , & LaCasse, C. F. (2017). Field deployable pushbroom hyperspectral imaging polarimeter. Optical Engineering, 56, 103107.

[pld3317-bib-0006] Lindqvist, K. (1960). Inheritance studies in lettuce. Hereditas, 46, 387–470. 10.1111/j.1601-5223.1960.tb03093.x

[pld3317-bib-0007] Mahlein, A.‐K. , Steiner, U. , Hillnhütter, C. , Dehne, H.‐W. , & Oerke, E.‐C. (2012). Hyperspectral imaging for small‐scale analysis of symptoms caused by different sugar beet diseases. Plant Methods, 8, 3. 10.1186/1746-4811-8-3 22273513 PMC3274483

[pld3317-bib-0008] Mo, C. , Kim, G. , Lim, J. , Kim, M. S. , Cho, H. , & Cho, B.‐K. (2015). Detection of lettuce discoloration using hyperspectral reflectance imaging. Sensors, 15, 29511–29534. 26610510 10.3390/s151129511 PMC4701346

[pld3317-bib-0009] Narayanasamy, P. (2011). Detection of fungal pathogens in plants. In P. Narayanasamy (Ed.), Microbial plant pathogens‐detection and disease diagnosis: Fungal pathogens (Vol. 1, pp. 5–199). Springer.

[pld3317-bib-0010] Ray, M. , Ray, A. , Dash, S. , Mishra, A. , Achary, K. G. , Nayak, S. , & Singh, S. (2017). Fungal disease detection in plants: Traditional assays, novel diagnostic techniques and biosensors. Biosensors & Bioelectronics, 87, 708–723. 27649327 10.1016/j.bios.2016.09.032

[pld3317-bib-0011] Rouse, J. W. (1974). Monitoring vegetation systems in the Great Plains with ERTS. In 3rd Earth Resource Technology Satellite (ERTS) Symposium (Vol. 1, pp. 48–62).

[pld3317-bib-0012] Sellar, R. G. , & Boreman, G. D. (2005). Comparison of relative signal‐to‐noise ratios of different classes of imaging spectrometer. Applied Optics, 44, 1614–1624. 15813264 10.1364/ao.44.001614

[pld3317-bib-0013] Simko, I. , Jimenez‐Berni, J. A. , & Furbank, R. T. (2015). Detection of decay in fresh‐cut lettuce using hyperspectral imaging and chlorophyll fluorescence imaging. Postharvest Biology and Technology, 106, 44–52.

[pld3317-bib-0014] Truco, M. J. , Ashrafi, H. , Kozik, A. , van Leeuwen, H. , Bowers, J. , Wo, S. R. C. , Stoffel, K. , Xu, H. , Hill, T. , Van Deynze, A. , & Michelmore, R. W. (2013). An ultra‐high‐density, transcript‐based, genetic map of lettuce. G3: Genes, Genomes, Genetics, 3, 617–631. 23550116 10.1534/g3.112.004929 PMC3618349

[pld3317-bib-0015] Villiger, M. , Pache, C. , & Lasser, T. (2010). Dark‐field optical coherence microscopy. Optics Letters, 35, 3489–3491. 20967109 10.1364/OL.35.003489

[pld3317-bib-0016] Wedge, D. E. , Smith, B. J. , Quebedeaux, J. P. , & Constantin, R. J. (2007). Fungicide management strategies for control of strawberry fruit rot diseases in Louisiana and Mississippi. Crop Protection, 26, 1449–1458.

[pld3317-bib-0017] Weßling, R. , & Panstruga, R. (2012). Rapid quantification of plant‐powdery mildew interactions by qPCR and conidiospore counts. Plant Methods, 8, 35. 10.1186/1746-4811-8-35 22937820 PMC3522566

[pld3317-bib-0018] Williamson, B. , Tudzynski, B. , Tudzynski, P. , & Van Kan, J. A. (2007). Botrytis cinerea: The cause of grey mould disease. Molecular Plant Pathology, 8, 561–580. 20507522 10.1111/j.1364-3703.2007.00417.x

[pld3317-bib-0019] Wu, D. , Feng, L. , Zhang, C. , & He, Y. (2008). Early detection of Botrytis cinerea on eggplant leaves based on visible and near‐infrared spectroscopy. Transactions of the ASABE, 51, 1133–1139.

